# Ocular hypertension suppresses homeostatic gene expression in optic nerve head microglia of DBA/2 J mice

**DOI:** 10.1186/s13041-020-00603-7

**Published:** 2020-05-25

**Authors:** James R. Tribble, Jeffrey M. Harder, Pete A. Williams, Simon W. M. John

**Affiliations:** 1 Department of Clinical Neuroscience, Division of Eye and Vision, St. Erik Eye Hospital, Karolinska Institutet, Stockholm, Sweden; 2 The Howard Hughes Medical Institute, The Jackson Laboratory, Bar Harbor, ME USA; 3 Department of Ophthalmology and Zuckerman Mind Brain Behavior Institute, Columbia University, New York, NY USA

**Keywords:** Glaucoma, Optic nerve head, Microglia, RNA-sequencing, DBA/2 J, Neuroinflammation, Mitochondria

## Abstract

Glaucoma is the leading cause of irreversible vision loss. Ocular hypertension is a major risk factor for glaucoma and recent work has demonstrated critical early neuroinflammatory insults occur in the optic nerve head following ocular hypertension. Microglia and infiltrating monocytes are likely candidates to drive these neuroinflammatory insults. However, the exact molecular identity / transcriptomic profile of microglia following ocular hypertensive insults is unknown. To elucidate the molecular identity of microglia after long-term exposure to ocular hypertension, we used a mouse model of glaucoma (DBA/2 J). We performed RNA-sequencing of microglia mRNA from the optic nerve head at a time point following ocular hypertensive insults, but preceding detectable neurodegeneration (with microglia identified as being CD45^lo^/CD11b^+^/CD11c^−^). Furthermore, RNA-sequencing was performed on optic nerve head microglia from mice treated with radiation therapy, a potent therapy preventing neuroinflammatory insults. Transcriptomic profiling of optic nerve head microglia mRNA identifies metabolic priming with marked changes in mitochondrial gene expression, and changes to phagocytosis, inflammatory, and sensome pathways. The data predict that many functions of microglia that help maintain tissue homeostasis are affected. Comparative analysis of these data with data from previously published whole optic nerve head tissue or monocyte-only samples from DBA/2 J mice demonstrate that many of the neuroinflammatory signatures in these data sets arise from infiltrating monocytes and not reactive microglia. Finally, our data demonstrate that prophylactic radiation therapy of DBA/2 J mice potently abolishes these microglia metabolic transcriptomic changes at the same time points. Together, our data provide a unique resource for the community to help drive further hypothesis generation and testing in glaucoma.

## Background

Glaucoma is one of the most common neurodegenerations affecting an estimated 80 million people worldwide [1]. It is a complex and multifactorial disease characterised by the progressive dysfunction and loss of retinal ganglion cells and their axons (that make up the optic nerve). A common theme between animal models and human glaucoma is the activation or reactivity of glial cells in the retina, optic nerve, and optic nerve head [2–7]. Activated microglia are known to affect the progression of neurodegenerative diseases due to their influence over homeostatic and immune responses. Ongoing microglial responses may be protective, damage neural tissue, and/or lead to chronic inflammation. Given the primary role of microglia in regulating neuroinflammation (such as in Alzheimer’s disease [8–11]), identifying any dysregulation of microglia is of paramount importance for the development of neuroprotective treatments [12].

Microglia perform a diverse series of functions to support neural activity, including maintenance of synapses and axons, removal of cellular debris, surveillance for injury and pathogens, and co-ordination of neuroinflammatory responses [12–17]. These functions require microglia to continuously sense and respond to their environment. Many environmental cues lead to changes in microglial gene expression that support functional specialization. Genome-wide gene expression profiling has been used to identify important functional differences between microglia in normal physiological, neuroinflammatory, and neurodegenerative conditions [18–20]. However, microglia-specific transcriptomic profiles in the normal and glaucomatous optic nerve head are yet to be generated. These previous studies support the need for transcriptomic profiling of microglia in glaucoma relevant tissues.

The DBA/2 J mouse develops neurodegeneration due to elevated intraocular pressure (IOP) with hallmark features of an inherited, chronic human glaucoma. An important, human relevant aspect of disease in DBA/2 J mice is elevated IOP-related injury at the optic nerve head (ONH). Elevated IOP is a major risk factor for human glaucoma. In our colony, elevated IOP develops from 6 months of age, and by 9 months of age there are signs of injury at the ONH [21, 22]. The neurodegeneration it triggers is complete in the majority of eyes by 12 months of age (based on retinal ganglion cell soma and axon loss) [22]. We have previously profiled genome wide gene expression in the whole ONH from 8.5 to 10.5 months of age to investigate disease progression due to elevated IOP before and during neurodegeneration [2, 3]. Notably these data pointed to major neuroinflammatory changes at a pre-degenerative stage of disease, including changes in the complement system, endothelin system, and cell-adhesion pathways. Our group and others have experimentally validated molecules in these pathways to demonstrate their importance and role in glaucoma (from mouse to human [2, 12, 23–29];). In addition, these pathways have been independently identified in other models of glaucomatous insult and retinal ganglion cell death (ocular hypertension, optic nerve crush, optic nerve transection / axotomy [30–32]) supporting the utility of DBA/2 J retina and optic nerve head tissue for modelling glaucoma. Despite the importance of neuroinflammation in DBA/2 J glaucoma, it remains unclear how microglia contribute to disease progression.

To investigate the role of microglia in the ONH in response to ocular hypertension, we performed RNA-sequencing of mRNA from optic nerve head microglia at a pre-degenerative stage of disease (9 months of age prior to detectable optic nerve degeneration). Furthermore, we performed transcriptomic profiling of microglia from radiation-treated mice, a potent and robust anti-inflammatory and neuroprotective therapy for DBA/2 J glaucoma [3, 33, 34]. Our results provide new information about potential dysfunction of these cells. We expect these data to be a novel resource for the glaucoma and neurodegeneration community.

## Methods

### Mouse strain, breeding and husbandry

Mice were housed and fed in a 14 h light / 10 h dark cycle with food and water available ad libitum; all mice used in the study were female. All breeding and experimental procedures were undertaken in accordance with the Association for Research for Vision and Ophthalmology Statement for the Use of Animals in Ophthalmic and Vision Research. The Institutional Biosafety Committee (IBC) and the Animal Care and Use Committee (ACUC) at The Jackson Laboratory approved this study. The DBA/2 J and DBA/2 J-*Gpnmb*^*R150X*^ (D2-*Gpnmb*^*+*^) strains were utilized and have been described in detail elsewhere [21]. Mice were used at 9 months of age when the majority of eyes have had ongoing IOP elevation but detectable neurodegeneration has yet to occur [2, 21, 35]. In DBA/2 J mice, mutations in two genes (*Gpnmb*^*R150X*^ and *Tyrp1*^*b*^) drive an iris disease with features of human iris atrophy and pigment dispersion. In this disease, pigment disperses from the iris and induces damage in the drainage structures of the eye. This inhibits aqueous humour outflow and leads to an increase in intraocular pressure [36]. We used D2-*Gpnmb*^*+*^ mice as a control, a non-glaucomatous substrain of DBA/2 J. For radiation treated DBA/2 J mice, mice were placed on a rotating platform and a sub-lethal dose of γ-radiation (7.5 Gy; D2-RAD) was administered using a ^137^Cesium source in a single dose at 10 weeks of age. Our previous data has demonstrated that this level of treatment does not cause any adverse conditions and does not require bone marrow reconstitution [3, 37]. The optic nerves of all mice used in this study were confirmed to have no detectable nerve damage or axon loss as assessed by PPD staining (*data not shown*).

### FAC sorting

**Fig. 1:**
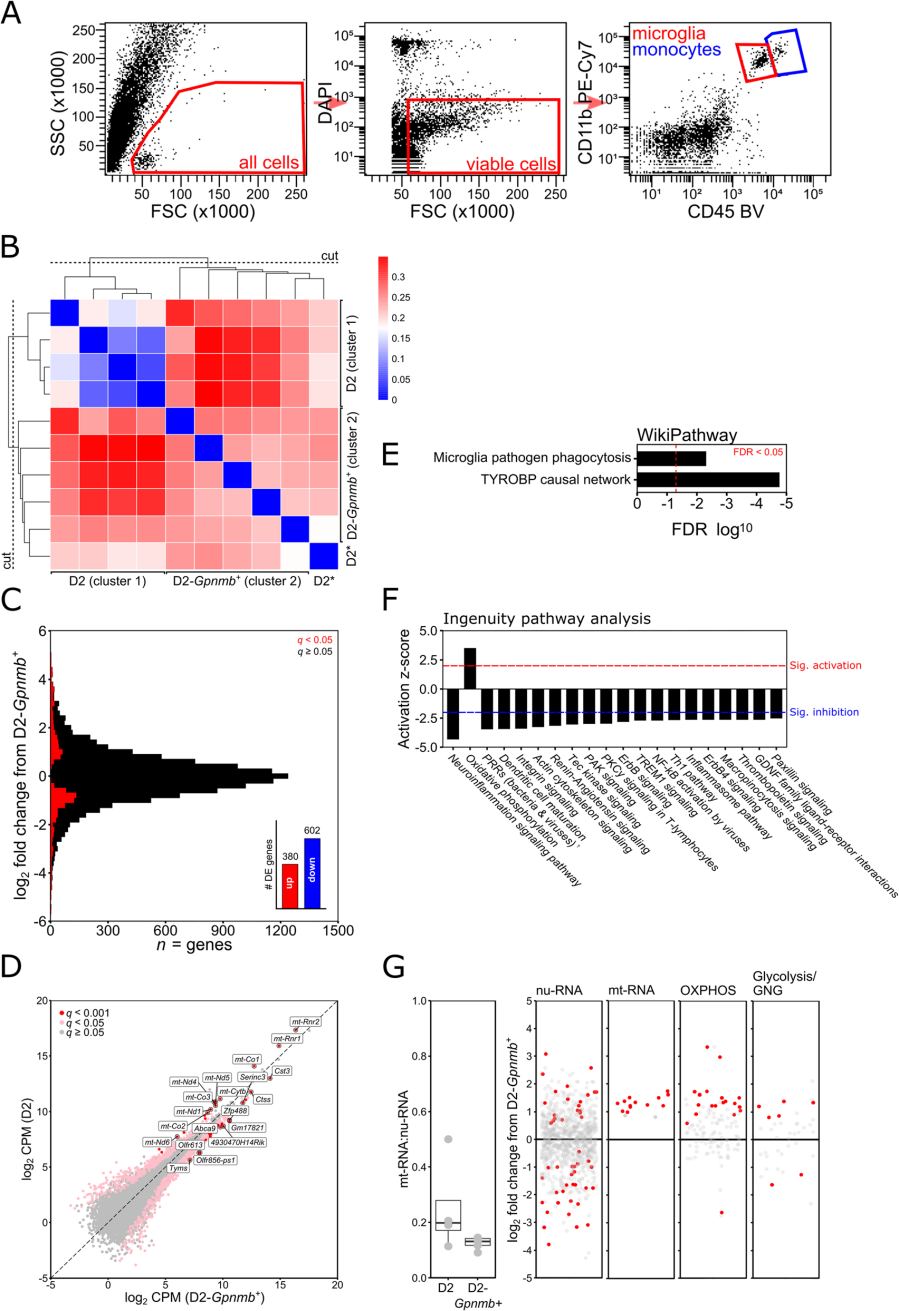
*RNA-sequencing of optic nerve head microglia from D2 and D2-Gpnmb*^*+*^*mice.* Microglia were FAC sorted from freshly isolated optic nerve heads and identified by being CD45^lo^/CD11b^+^ (**a**, see also Methods). Following RNA-sequencing of microglia from D2 and D2-*Gpnmb*^*+*^ optic nerve heads, samples were grouped by unsupervised hierarchical clustering (**b**; blue = strong correlation, red = weak correlation), creating D2 and D2-*Gpnmb*^*+*^ clusters (* denotes outlier excluded from subsequent analysis). **c** Genes were binned by log_2_ fold change (bin width 0.2) and coloured to show DE genes (red; *q* < 0.05). A simple summary is shown in the inset of **c**. **d** Scatter plot of all genes by mean log_2_ counts per million (CPM) for D2 (*y*) against D2-*Gpnmb*^*+*^ (*x*) showing DE genes (pink, *q* < 0.05; red, *q* < 0.001; non DE genes in grey) with top 20 DE genes annotated. Pathway analysis of DE genes revealed dysregulation of pathways involved in the microglial sensome and metabolism (**e** and **f**. Wikipathway analysis (**e**) showed significant dysregulation of 2 inflammatory pathways (*q* < 0.05). The number of DE genes within each pathway is shown . Ingenuity pathway analysis (**f**) also showed dysregulation of a number of sensome and inflammatory pathways, and metabolism pathways. Top 20 pathways sorted by *z*-score are shown, with the threshold for significant activation or inhibition marked. We queried metabolism dysregulation (**g**) demonstrating a trend towards an increased ratio of mt-RNA:nu-RNA in D2 microglia (*P* = 0.23) and DE gene expression (red, *q* < 0.05) in mitochondrial transcripts from nu-RNA and mt-RNA. DE of genes involved in OXPHOS and glycolysis/gluconeogenesis (GNG). In **f**^†^PPRs = pattern recognition receptors

FAC sorting of cells from the optic nerve head was performed as previously described [37]. Prior to cell collection, all surfaces and volumes were cleaned with 70% ethanol and RNaseZap (ThermoFisher Scientific) solution followed by dH_2_0. Mice were euthanized by cervical dislocation, eyes enucleated, and placed immediately into ice-cold HBSS. For single ONH isolation, eyes were enucleated from the globe with curved scissors and the eye placed directly into ice cold HBSS (Gibco). The eye was punctured at the limbus with a 28 G needle and the cornea, iris, and lens removed to leave a posterior eye cup. A single cut was made through the choroid to the tissue surrounding the ONH, and the ONH (including the central retina and some extraocular tissue and choroid) was removed free from the globe. The ONH, representing the transparent pre-myelin transition zone segment of the optic nerve (~ 500 μm in length), was separated from the central retina and myelinated optic nerve and any additional pigmented tissue/extraocular tissue was removed. Single ONHs were placed directly into 100 μl of a custom HBSS, dispase (5 U/ml) (Stemcell Technologies), DNase I (2000 U/ml) (Worthington Biochemical), and SUPERase (1 U/μl) (ThermoFisher Scientific) solution. Samples were incubated for 20 mins at 37 °C and shaken at 350 RPM in an Eppendorf Thermomixer R followed by gentle trituration using a 200 μl pipette. Samples were blocked in 2% BSA, SUPERase (1 U/μl) in HBSS, and stained with secondary conjugated antibodies against CD11b, CD11c, CD34, CD45.2, GFAP (detailed in Table 1), as well as DAPI. This cocktail allowed other cell types to be accurately removed during FACS. FACS was performed on a FACSAria (BD Biosciences). Singlet gating of SSC-H vs SSC-W and FSC-H vs FSC-W gating plots was used to identify single cells. FSC-H vs DAPI was used to identify viable cells and CD11b^+^/CD45.2^lo^ (and negative for all other markers; Fig. 1a) microglia were sorted into 100 μl buffer RLT + 1% β-ME, vortexed and frozen at − 80 °C until further processing. We have previously performed RNA-sequencing on mRNA of infiltrating monocytes (CD45^hi^/CD11b^+^/CD11c^+^) from the optic nerve head of 9 month DBA/2 J mice [37]. This marker panel was based on our previous findings that identified the majority of infiltrating immune cells as CD11c^+^ [3]. In these previous flow cytometry experiments < 3% of all myeloid derived cells (CD45^+^/CD11b^+^) in 9 month of age DBA/2 J optic nerve head tissue were resident CD11c^+^ microglia (CD45^lo^/CD11b^+^) [3, 37], and thus, CD11c^+^ microglia make up a negligible proportion of optic nerve head immune cells at this time point. In the current study, we aimed to enrich for resident microglia, as opposed to microglia that may be derived from infiltrating immune cells, and enriched for CD11c^−^ microglia for this purpose.

**Table 1:** Flow cytometry antibody details

Antibody	Fluorophore	Source
Anti-mouse CD11b (clone M1/70)	PE-Cy7	BD Biosciences, Cat # 552850
Anti-mouse CD11c (clone N418)	PE	Tonbo Biosciences, Cat # 50-0114-U025
Anti-mouse CD34 (clone RAM34)	APC	Thermo Fisher, Cat # 50-0341-82
Anti-mouse CD45.2 (clone 104)	Brilliant Violet 421	Biolegend, Cat # 109831
Anti-human GFAP	AF488 (pre-conjugated secondary)	Abcam, Cat # ab4674

### RNA-sequencing and analysis

Microglia samples were defrosted on ice and homogenized by syringe in RLT Buffer (total volume 300 μl). Total RNA was isolated using RNeasy micro kits as according to manufacturer’s protocols (Qiagen) including the optional DNase treatment step, and quality was assessed using an Agilent 2100 Bioanalyzer. The concentration was determined using a Ribogreen Assay from Invitrogen. Amplified dscDNA libraries were created using a Nugen Ovation RNA-seq System V2 and a primer titration was performed to remove primer dimers from the sample to allow sample inputs as low as 50 pg RNA. The SPIA dscDNA was sheared to 300 bp in length using a Diogenode Disruptor. Quality control was performed using an Agilent 2100 Bioanalyzer and a DNA 1000 chip assay. Library size produced was analysed using qPCR using the Library Quantitation kit/Illumina GA /ABI Prism (Kapa Biosystems). Libraries were barcoded, pooled, and sequenced 6 samples per lane on a HiSeq 2000 sequencer (Illumina) giving a depth of 30–35 million reads per sample.

Following RNA-sequencing samples were subjected to quality control analysis by a custom quality control python script. Reads with 70% of their bases having a base quality score ≥ 30 were retained for further analysis. Read alignment was performed using TopHat v 2.0.7 and expression estimation was performed using HTSeq with supplied annotations and default parameters against the DBA/2 J mouse genome (build-mm10). Bamtools v 1.0.2 were used to calculate the mapping statistics. Differential gene expression analysis between groups was performed using edgeR v 3.10.5 [38, 39] and the removal of outlier samples and lowly expressed genes was achieved by removing genes at a pre-defined cut-off level. We only included genes that were expressed at > 1 counts per million (CPM) in ≥4 samples across all samples for D2-*Gpnmb*^*+*^ to DBA/2 J (D2) comparison and ≥ 3 samples across all samples for D2 to D2-RAD comparison (chosen based on the size of the smallest group). We used unsupervised hierarchical clustering (HC) to generate clusters of samples with distinct gene expression profiles in which as many control samples were represented in a single cluster. HC was performed in R (1-cor, Spearman’s *rho*) based on a matrix of all samples, representing all genes post cut-off. Clusters were required to have ≥3 samples in order to compare using statistical testing; clusters that did not meet these criteria were removed from the analysis as outliers. (Although D2 sample D2_S1 was more distant than others within its cluster, analysing the data without this sample made no changes to the outcomes, *data not shown*.) Adjustment for multiple testing was performed using false discovery rate. DEseq2 and limma were used for differential expression analysis [40, 41]. Genes were considered to be significantly differentially expressed at a false discovery rate (FDR; Benjamini and Hochberg adjusted *p* values; *q*) of *q* < 0.05. Pathway analyses (see Results) were performed in R, WebGestalt (www.webgestalt.org; provides continuously curated, publicly availably pathways for exploring pathway enrichment) [42], and Ingenuity pathway analysis (IPA, Qiagen; which further explores directionality of pathway enrichment). Mitochondria gene lists were taken from published lists [43, 44]. Graphing was performed in R. Complete raw untrimmed count files can be found in Supplementary Data 1.

For comparisons to other published datasets, the microglia dataset generated here was compared to DBA/2 J whole optic nerve head at 8.5 months of age [2] (publically available from Datgan [45]) and DBA/2 J optic nerve head monocytes at 9 months of age [37] (Supplementary Data 2).

For the Supplementary Data 3 and 4, *t* refers to the *t* statistic, and B refers to log-odds that the gene is differentially expressed.

## Results

### Transcriptomic profiling of optic nerve head microglia

We performed RNA-sequencing on CD45^lo^/CD11b^+^/CD11c^−^ microglia samples from DBA/2 J (D2), control D2-*Gpnmb*^*+*^, and radiation treated DBA/2 J (D2-RAD) mice isolated from single optic nerve heads (ONHs) and enriched through FAC sorting (see Methods, and Fig. 1a). A total of 15 samples were amplified and sequenced (*n* = 5 for all groups; cell inputs = 165 ± 37 for D2, 182 ± 145 for D2-*Gpnmb*^*+*^, and 162 ± 173 for D2-RAD). To confirm the isolated cells were microglia, genes known to be highly expressed in microglia, astrocytes, neurons, oligodendrocytes, and infiltrating monocytes were analysed (Table 2). Genes associated with microglia were highly expressed in these samples. Low to no expression of other cell-type specific genes was observed, consistent with the samples primarily containing microglia.

**Table 2:** Cell type specific gene expression

Cell type	Gene	Average expression (log_2_ CPM) ^a^
Myeloid	*Tmem119*	9.53
Myeloid	*P2ry12*	9.93
Myeloid	*Siglech*	8.53
Myeloid	*Gpr34*	7.79
Myeloid	*Olfml3*	9.06
Myeloid	*Cx3cr1*	10.88
Myeloid	*Aif1* (Iba1)	7.23
Myeloid (activated)	*Itgax*	4.66
Myeloid (activated)	*Ccr2*	3.73
Myeloid (activated)	*Ly6c1*	0.94
Myeloid (activated)	*Mrc1* (CD206)	4.89
Myeloid (activated)	*Cd68*	8.60
Astrocyte	*Gfap*	0.80
Astrocyte	*Gjb6*	N.D
Astrocyte	*Ntsr2*	N.D
Astrocyte	*Aldh1l1*	−0.50
Astrocyte	*Aldoc*	N.D
Astrocyte	*Aqp4*	0.38
Oligodendrocyte	*Mobp*	N.D
Oligodendrocyte	*Mog*	N.D
Oligodendrocyte	*Cldn11*	N.D
Oligodendrocyte	*Plp*	N.D
Neuronal	*Tubb3*	N.D
Neuronal	*Vglut1*	N.D
Neuronal	*Rpbox3* (NeuN)	N.D
Neuronal	*Syt1*	1.75
Neuronal	*Stmn2*	N.D
Neuronal	*Snap25*	3.44
Neuronal	*Eno2*	2.52
Neuronal	*Syn1*	N.D
Housekeeping	*Actb*	12.09
Housekeeping	*Gapdh*	9.35

*N.D* not detected (below cut-off), ^a^ average across all D2 and D2-*Gpnmb*^*+*^ samples

Differences between D2 samples were expected due to the spontaneous nature of IOP insults, with some samples still resembling controls [35]. Hierarchical clustering (HC) [2, 28] demonstrated that the majority of D2 samples were more similar to each other than to normotensive D2-*Gpnmb*^*+*^ samples. HC generated two major clusters of samples containing: (1) four D2 samples (D2_S1–4), (2) five D2-*Gpnmb*^*+*^ samples (D2Gpnmb_S1–5) and one D2 sample (D2_S5) (Fig. 1b). The D2 sample in the second cluster (D2_S5) was removed for further analyses to create distinct disease (cluster 1; *n* = 4) and control (cluster 2; *n* = 5) groups. Between groups, 982 genes were differentially expressed (DE, Fig. 1c and d, Supplementary Data 3). Functional analysis of DE genes revealed significant changes in pathways associated with microglial function including surveillance, phagocytosis, metabolism, and inflammation (Fig. 1e and f), which are further expanded on below.

### Genes that regulate microglial surveillance and phagocytosis are downregulated by chronic IOP elevation

A majority of the DE genes were downregulated in microglia from D2 eyes (Fig. 1c). These downregulated genes contributed to the enrichment of the TYROBP causal network and microglial pathogen phagocytosis pathways (Fig. 1e). The TYROBP causal network links the activation of TREM2 receptors with gene expression and thus contributes to the overall state of microglial activation. The downregulation of numerous genes in the network is consistent with a decrease in TYROBP signalling [46]. Ingenuity Pathway Analysis (IPA) identified another 19 pathways predicted to be significantly inhibited or less active in D2 microglia compared to D2-*Gpnmb*^*+*^ microglia (Fig. 1f). These pathways span a wide range of functions including phagocytosis, cell movement and shape, receptor-mediated signalling, and inflammation. Common downregulated DE genes within these pathways included C1 complex encoding genes (*C1qa*, *C1qb*, *C1qc*), integrins (*Itgam*, *Itgb2*, *Cd37*), Ig superfamily (*Trem1*, *Trem2*, *Il10ra*, *Il13ra*), phagocytic components (*Nckap1l*) and toll-like receptor signalling (*Tlr1*, *Tlr3*, *Tlr7*, and toll-like receptor pro-inflammatory enhancer *Themis2* [47]). These data predict that many functions of microglia that help maintain tissue homeostasis are affected, and potentially inhibited, by chronic ocular hypertension.

### Metabolism-related transcripts are upregulated in microglia by chronic IOP elevation

Changes in genes with mitochondrial and metabolic functions were identified in both gene and pathway level analyses. Of the top 20 DE genes (sorted by *q*) in D2 microglia compared to D2-*Gpnmb*^*+*^, 10 were mitochondrial transcriptome derived (mt-RNA, Fig. 1d). Nuclear encoded mitochondrial transcripts (nu-RNA) also differed from controls (22 up, 29 down, Fig. 1g). Ingenuity pathway analysis predicted that these changes promote oxidative phosphorylation (OXPHOS) activity (Fig. 1f). 17.5% of OXPHOS genes were DE and 19/20 DE genes had higher expression in D2 mice (Fig. 1g). Changes in the ratio of mt-RNA:nu-RNA can indicate changes in intracellular signalling between the mitochondria and nucleus. This ratio showed high variation between D2 samples (0.25 ± 0.17) but the mean ratio was not significantly different from D2-*Gpnmb*^*+*^ samples (0.13 ± 0.02; *P* = 0.23, *Student’s t-*test, Fig. 1g).

To further understand the metabolic state of microglia, we considered additional changes in metabolic genes. *Hif1a*, a master regulator of glycolysis and cell stress responses [48], was upregulated consistent with stress or inflammation, and with previous findings in the inner retina during glaucoma [35, 49]. Glycolysis regulator *Pfkfb2* [50] was upregulated, as well as other glycolysis genes (*Gapdh*, *Pgam1*, *Pgk1*, *Pgm2l1*) (Fig. 1g). *Slc16a1* (MCT1) was also up-regulated suggesting increased lactate, pyruvate, or ketone bodies transport [51]. The transporter is bi-directional, and as such could reflect either an attempt to increase microglial energy sources, or to increase metabolic support to retinal ganglion cell axons in the optic nerve head. Taken together, these data suggest that optic nerve head microglia in D2s develop an increased capacity to metabolise energy from various sources.

Metabolic switching between oxidative phosphorylation and glycolysis occurs in disease when microglia transition between pro-inflammatory and anti-inflammatory states. This cellular transition is associated with gene expression changes induced by both ROS and cytokine signalling pathways. In optic nerve head microglia, no enrichment was observed in pro or anti-inflammatory pathways based on the number of DE genes (Fig. 1e). Based on the direction of expression changes, three pro-inflammatory pathways were predicted to be inhibited; neuroinflammation signalling, Th1 signalling, and inflammasome signalling (Fig. 1f). We also assessed changes in a list of 20 genes associated with canonical M1 and M2 inflammation phenotypes [52]. Only two genes were differentially expressed, *Ccl5* (decreased) and *Tgfb2* (increased). TGF-β is an important anti-inflammatory signal for microglia that regulates their morphology, proliferation, and survival [53]. TGF-β signalling has been implicated in both homeostatic and anti-inflammatory signalling in microglia. Overall microglia gene expression in response to chronic IOP elevation was not pro-inflammatory, although they are predicted to be primed metabolically to facilitate rapid changes in phenotype.

### Microglia and infiltrating monocytes have distinct phenotypes in pre-degenerative tissue

**Fig. 2:**
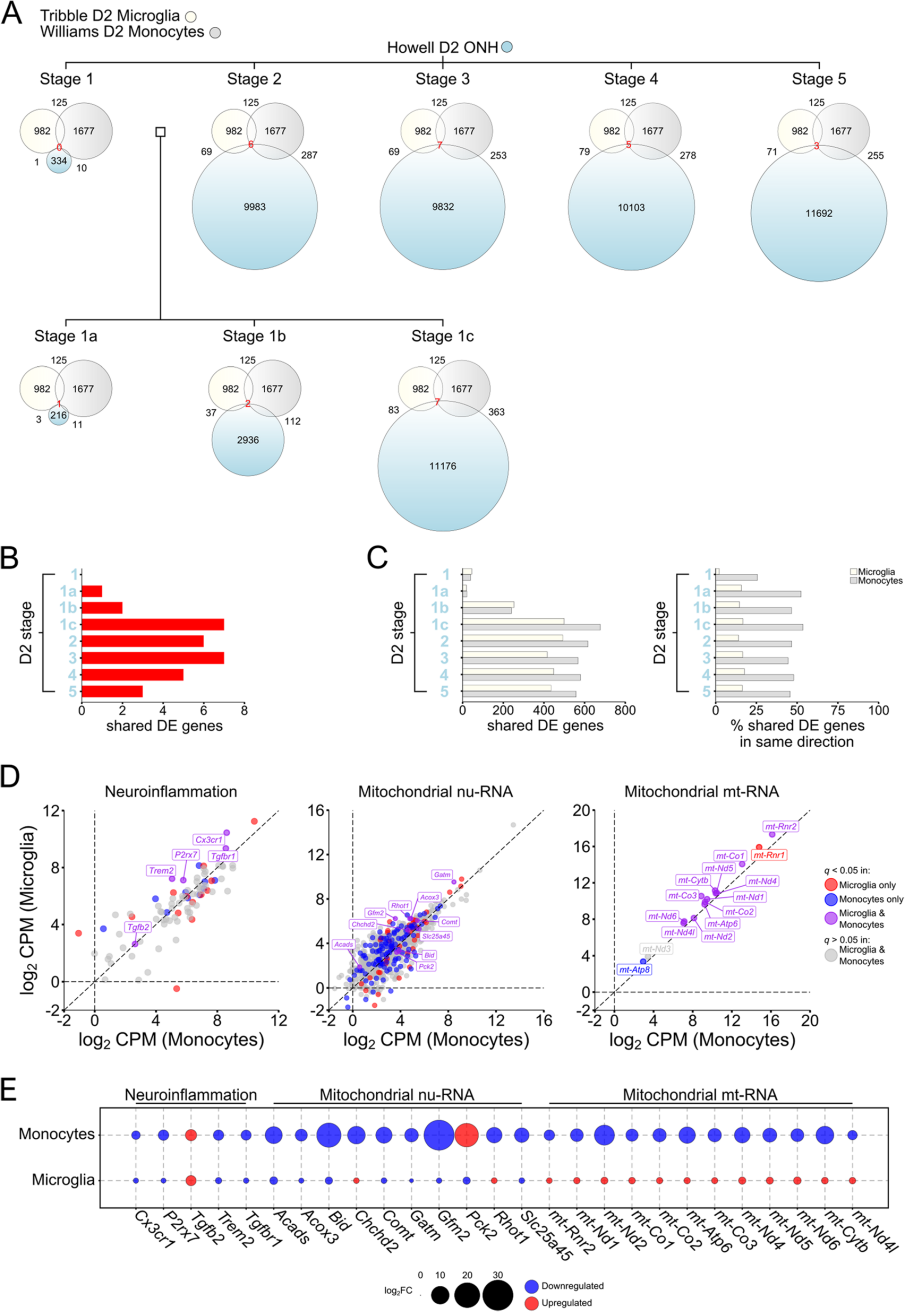
*Monocytes, not microglia, are likely drivers of inflammatory signatures in glaucoma gene expression datasets.* Comparison between DE genes in microglia (Tribble; current dataset), monocytes (Williams [37];), and whole optic nerve head in the D2 (Howell [2, 3];). Howell et al. [2] previously identified 5 molecularly distinct stages of disease in the D2 whole optic nerve head, where stages 1–2 show no morphologically detectable neurodegeneration. Further study subdivided stage 1 into 3 stages (1a, 1b, and 1c as shown between stages 1 and 2 here) [3]. **a** Euler diagrams show total number of DE genes within each dataset (number within each circle), the number of shared DE genes (with matching upregulation or downregulation, shown outside the corresponding intersection, and centrally (in red text) for matching genes for all 3 datasets (also displayed in **b**). **c** When direction of change is not considered, there are a greater number of shared DE genes for both microglia and monocytes, but a greater percentage of these are of matching upregulation or downregulation in monocytes. This may reflect strength of contribution of monocytes to whole optic nerve head changes over microglia. Further comparisons of neuroinflammatory genes (based on IPA gene sets) or mitochondria-related transcripts (from MitoCarta [43, 44]) demonstrate a number of genes that are uniquely DE in either microglia or monocytes (**d**; red = DE in microglia, blue = DE in monocytes; log_2_ CPM from D2 microglia and D2 infiltrating monocytes). Of the few shared DE genes (**d**; purple) the majority of neuroinflammatory and nu-genes show the same directional changes but the opposite was true for mt derived genes (**e**; upregulation = red, downregulation = blue)

We compared gene expression changes in the isolated D2 microglia to previously defined gene expression changes from whole ONH tissue [2, 3] and from infiltrating monocytes [37] both from D2 mice at similar ages (Fig. 2, Supplementary Data 2). Howell et al. previously identified 5 molecularly distinct stages of disease in the D2 whole optic nerve head using hierarchical clustering of RNA microarray data, where stages 1–3 show no morphologically detectable neurodegeneration. A further 3 early stages (between stage 1 and 2) were subsequently identified [2, 3]. Based on corresponding upregulation or downregulation of genes, there was partial overlap between expression changes in isolated D2 microglia and each staged group of ONH samples (Fig. 2a). The most overlap with the D2 ONH was at an ocular hypertensive and pre-degenerative stage, representing 0.7% of DE genes in the ONH and 8% in microglia. A similar comparison between infiltrating monocytes and the ONH showed greater overlap, with 4-fold more genes in common. Very few DE genes showed the same directional DE changes across all 3 groups (Fig. 2b). A comparison irrespective of directional regulation showed a larger number of common DE genes between either microglia or monocytes with whole optic nerve head tissue (Fig. 2c), possibly as cell-specific effects may be masked in whole tissue analysis. Taken together, these data suggest that these two myeloid-cell derived populations have largely distinct responses relative to whole tissue changes in the ONH after chronic ocular hypertension.

To further elucidate the neuroinflammatory and metabolic phenotypes, we compared neuroinflammatory and mitochondria gene expression in microglia and monocytes (Fig. 2d and e). Of 144 neuroinflammatory genes annotated by Ingenuity, few were DE in either microglia (*n* = 18) or monocytes (*n* = 16) at this pre-degenerative stage of disease (Fig. 2d). The shared neuroinflammatory DE genes (*n* = 5) showed the same direction of change in both cell types (Fig. 2e). These five genes (*Cx3cr1*, *Tgfb2*, *Tgfbr1*, *Trem2*, and *P2rx7*) offer excellent candidates for genetic manipulation to test function of innate immune pathways in the optic nerve head.

Metabolic changes are a prominent feature in cells and tissue affected by elevated IOP based on RNA-sequencing datasets [35, 37, 54]. Changes to transcripts encoding mitochondrial proteins feature in both the microglia and monocyte RNA-sequencing datasets (Fig. 2d and e). Dysregulation of nu-RNA was greater in monocytes (*n* = 121 genes) than microglia (*n* = 48 genes). Of the 10 shared DE nu-genes, 7 showed the same directional change (Fig. 2e) but with a greater magnitude of dysregulation in monocytes. For mt-RNA transcripts 12/15 genes were DE in both monocytes and microglia, but with markedly different expression profiles (0/12 being co-up- or co-down- regulated; Fig. 2e). Thus, our data suggest a pro-metabolic status in microglia that is not matched in infiltrating monocytes. Together our data implicate mitochondrial / metabolic changes in optic nerve head immune cells as an early disease feature.

### Pre-treatment by irradiation reduces the effects of ocular hypertension on microglia

**Fig. 3:**
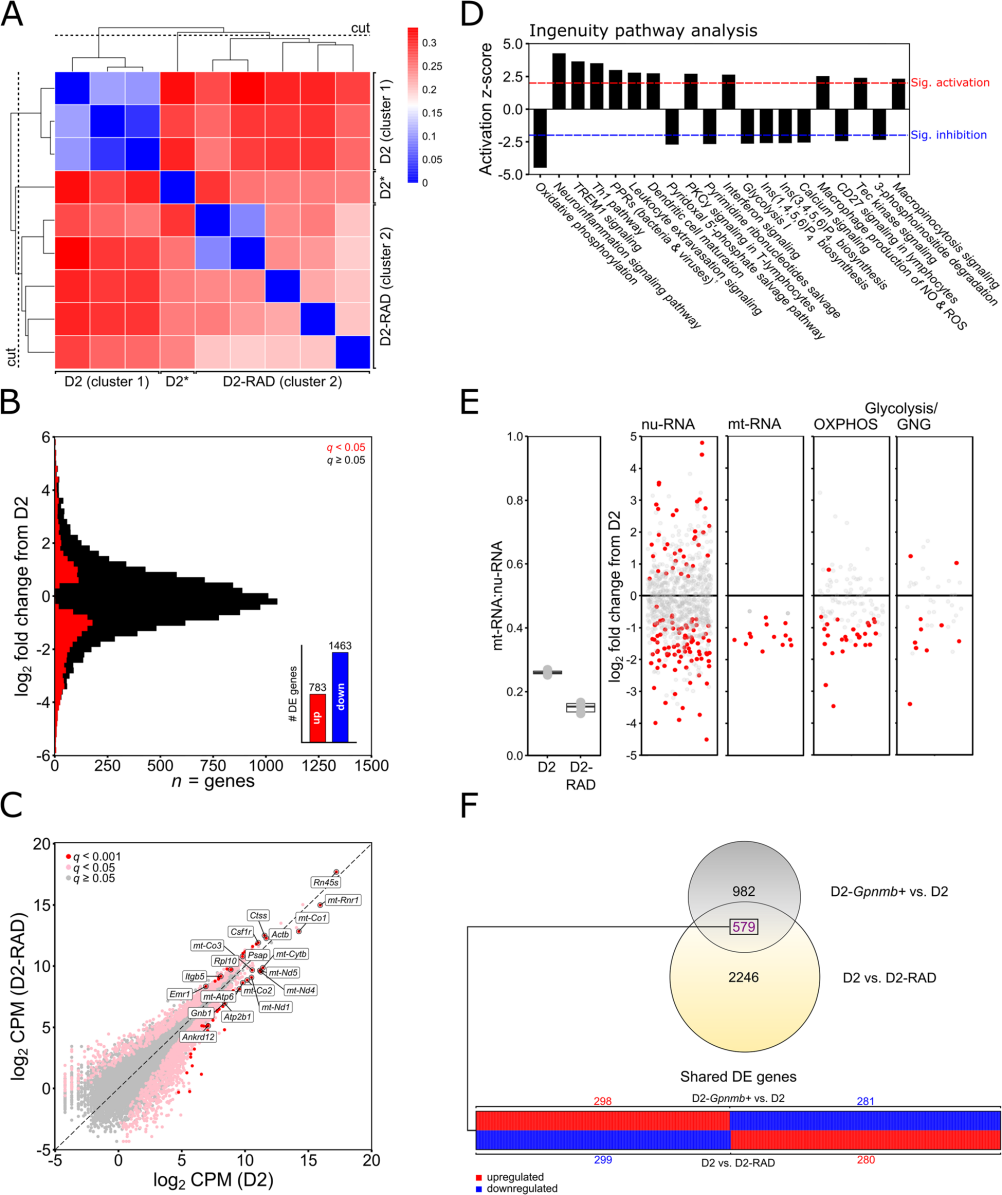
*RNA-sequencing of optic nerve head microglia from D2 and radiation therapy treated D2 mice.* Following RNA-sequencing of microglia form D2 and radiation therapy treated (RAD-D2) optic nerve heads, samples were grouped by unsupervised hierarchical clustering (**a**; blue = strong correlation, red = weak correlation), creating D2 and RAD-D2 clusters (* denotes outlier excluded from subsequent analysis). **b** Genes were binned by log_2_ fold change (bin width 0.2) and coloured to show DE genes (red; *q* < 0.05). A simple summary is shown in the inset of **b**. (**c**) Scatter plot of all genes by mean log_2_ counts per million (CPM) for D2 (*x*) against RAD-D2 (*y*), showing DE genes (pink, *q* < 0.05; red, *q* < 0.001; non DE genes in grey) with top 20 DE genes annotated. Ingenuity pathway analysis (**d**) showed dysregulation of a number of sensome and inflammatory pathways, and metabolism pathways, representing a correction of the D2 phenotype in RAD treated animals. Top 20 pathways sorted by *z*-score are shown, with the threshold for significant activation or inhibition marked. Relevant to metabolism and signaling (**e**) mt-RNA:nu-RNA ratios are shown. DE gene expression (red, *q* < 0.05) in mitochondrial transcripts in nu-RNA and mt-RNA are shown. Genes involved in OXPHOS and glycolysis/gluconeogenesis (GNG) are compared. **f** Overlap of DE genes from D2 vs. D2-*Gpnmb*^*+*^ and D2 vs. RAD-D2 datasets. In **e**^†^PPRs = pattern recognition receptors

Low dose irradiation of mice at a young age prevents glaucomatous neurodegeneration in D2 mice without lowering IOP [3]. Altered microglia have been suggested to contribute to the protective effects of radiation [34]. We compared and analysed D2 against RAD-D2 samples. HC generated 2 clusters representing 1) three D2 samples (D2_S1, 2, and 4), and 2) five RAD-D2 samples (D2-RAD_S1–5) and 1 D2 sample (D2_S3) (Fig. 3a). This single D2 sample from cluster 2 was removed by the dendogram cut (Fig. 3a). RAD-D2 microglia exhibited 2246 DE genes compared to D2 microglia (783 upregulated, 1463 downregulated) (Fig. 3b and c, Supplementary Data 4). Pathway analyses (Fig. 3d-f) showed that radiation treatment affects phagocytosis, metabolism, mitochondria, and inflammation related genes, all pathways altered in microglia (*see above*). There were 579 genes altered by radiation that overlapped with changes in glaucoma (D2 vs. D2-*Gpnmb*^*+*^comparison). For 578 of these genes, radiation treatment corrected the disease-related change (Fig. 3f). *Dcbld2* was the single gene DE downregulated in both datasets. *Dcbld2* encodes the endothelial and smooth muscle cell-derived neuropilin-like protein (ESDN) which is upregulated in endothelial cells following vascular injury [55]. Its deletion or downregulation impairs retinal angiogenesis [56] and promotes insulin signalling [57]. Its expression is not limited to endothelial cells; with relatively robust expression in microglia, astrocytes, and neurons (brain RNA-sequencing [58]).

## Discussion

Neuroinflammation at the site of the optic nerve head (ONH) may be a critical pathogenic event in glaucoma. Our group and others have identified the ONH as a likely candidate for the initial site of damage in glaucoma across species [22, 59–61]. In this manuscript we have identified further transcriptomic changes at the level of ONH microglia. The current study focuses on CD11c^−^ microglia, the most prominent microglia subtype in our previous datasets (representing > 97% of microglia identified in the ONH, [3, 37]). We have previously used CD11c as a marker to distinguish myeloid-derived cell subtypes within the ONH; with the majority of infiltrating monocyte-like cells in the ONH being CD45^hi^ and CD11c^+^. Emerging evidence is demonstrating a role for CD11c^+^ microglia in neurodegenerative disease progression, especially during demyelinating events [62], and with T-cell interactions in the brain [63]. Given that the ONH is unmyelinated, and that T-cell changes have not been found in the glaucomatous ONH, it is unsurprising to observe so few CD11c^+^ microglia. Here, we focused on Cd11c^−^ microglia, a sub-type more associated with tissue surveillance and inflammation. These microglia were affected by chronic elevated intraocular pressure (IOP) based on changes at a transcriptional level, consistent with previous studies showing microglial activation in glaucoma.

To explore molecular changes that lead to optic nerve degeneration we have previously performed microarray gene expression profiling of the whole ONH [2, 3]. This data set shows changes to inflammatory pathways, but the attributive cells were unknown. To further understand the molecular events that happen in the ONH at a cell-type level we have performed transcriptomic profiling of monocyte-like cells in the same model of glaucoma [37]. These cells were highly pro-inflammatory and express various complement genes and integrins. Targeting the α subunit of complement receptor 3 (genetic ablation of *Itgam* encoding CD11b) prevents monocyte-like cell extravasation into the ONH and significantly reduced the risk of developing severe glaucomatous neurodegeneration. CD11b is well expressed on microglia [64] and we used cell-surface expression of CD11b to enrich for microglia. In the data presented here we predict that DBA/2 J microglia are initially anti-inflammatory. *Itgam* is downregulated in DBA/2 J microglia (in this data set) following periods of ocular hypertension. Nevertheless, the protection that results from removing CD11b (*Itgam* knockout [37];) could, in part, be due to its effects on microglia, but elucidating exactly which cell-type is at play will take definitive testing using cell-type-specific *cre*-lines. This dichotomy of microglia and monocytes in the ONH offers an exciting avenue for further research, particularly during the stage of monocyte extravasation into the ONH. For example, microglia processes have been shown to reseal injured capillaries following recruitment via P2ry12 mediated chemotaxis [65]. *P2ry12* is downregulated in D2 microglia in the present study, which could suggest defects in initial-protective microglial responses that exacerbate monocyte initiated injury.

To date, one of the most protective therapies in DBA/2 J glaucoma has been radiation therapy [3, 6, 33, 34]. A sub-lethal dose of radiation (γ- or X- ray) early in life changes the neuroinflammatory response to ocular hypertension later in life. One reason that radiation treatment is protective is that it increases the expression of GlyCAM1 in the retina and ONH, thereby reducing the entry of pro-inflammatory monocytes [6]. Another type of neuroinflammatory cell-type affected by radiation therapy is microglia [34]. Microglia respond acutely to radiation therapy; however, the long-term effects on microglia need to be more fully defined. As presented here, the majority of genes (59%) dysregulated by elevated IOP in untreated DBA/2 J microglia had a different level of expression in D2-RAD microglia. In addition, microglia in treated mice had a different predicted activation state of TREM1 signalling and more broadly alterations of neuroinflammation signalling and metabolic pathways related to energy production (Fig. 3d). It is not clear yet whether this is a direct effect of the radiation treatment within microglia or a secondary effect of microglial interactions with other cells. TREM1 signalling may be a critical factor in controlling microglial activation in glaucoma and needs to be tested further. Determining the function of TREM1 signalling in glaucoma is likely to uncover more specific mechanisms of damage by elevated IOP and protection by radiation therapy.

Phagocytosis is suggested to be a critical process in the ONH for healthy tissue maintenance [66], but its role in ONH microglia has not been explored. Microglial phagocytosis is generally regulated by the TREM2-TYROBP signalling pathway [67], and the TYROBP signalling network in microglia is among the earliest affected by chronic elevated IOP. Mutations in *TYROBP* cause Nasu-Hakola disease [68], in which the neurodegenerative pathology is defined as a primary microglial disorder [69]. This underlies the important function of this pathway and microglia toward directly preventing disease. Disruption of the TYROBP signalling network has also been demonstrated in Alzheimer’s disease [46]. Furthermore, the increased risk of Alzheimer’s disease caused by *TREM2* mutations [70] has led to research showing many effects of TREM2 on phagocytosis, transcription, metabolism, and inflammation [71–73]. These data suggest that this signalling pathway may be a master regulator of other changes observed here. Manipulating TYROBP and TREM2 is a promising strategy to define functions of microglia in glaucoma and a possible avenue for treatment.

Retinal ganglion cell axons remain unmyelinated at the ONH and therefore are particularly vulnerable to glaucoma related stresses (age and elevated intraocular pressure) in addition to metabolic strain. Recently we have demonstrated that a critical metabolic vulnerability exists in retinal ganglion cells in DBA/2 J glaucoma with marked mitochondrial and metabolic changes [35]. Preventing these metabolic events (either systemically or specific to retinal ganglion cells) robustly protects from glaucomatous neurodegeneration [25, 54] although neuroinflammatory features still remain [25]. The neuro-glia-vascular complexes of the ONH form a contained metabolic unit in which glia provide trophic and metabolic support to retinal ganglion cell axons [74]. In our data, microglia are predicted to become more metabolically active, a metabolic shift that is typically anti-inflammatory and pro-supportive to neurons [75]. In this sense, microglia could be early mediators of neuroprotection in glaucoma. More work is needed to explore microglial function at different stages of disease, along with a consideration of microglial subtypes. One subtype, CD11c^+^ microglia, represent a small proportion of ONH microglia in our early-stage data, but may become important at later stages or in other regions of affected tissue. It is also possible that microglia later become pro-inflammatory. Thus further experiments are needed to resolve how elevated IOP triggers changes in microglia and if polarization of microglia continues to change during disease progression.

Metabolic treatments that protect retinal ganglion cells do not prevent all neuroinflammatory events in the retina and ONH [25, 35]. The long-term effects of this neuroinflammation on survival or function of the optic nerve are not known. Thus, inflammation remains an important target to consider for new therapies. At this pre-degenerative stage of glaucoma, we observed few changes in inflammatory molecules in microglia, and attribute many of the neuroinflammatory changes to infiltrating monocyte-like cells. Recent evidence using single cell RNA-sequencing has demonstrated that microglia display a heterogeneous repertoire of inflammatory responses in diseased tissue. Our study may lack the cellular resolution to detect microglial heterogeneity associated with inflammation, if present. However, reactive microglia in the ONH have been identified immunohistochemically in glaucoma [5, 7] and more precisely characterizing the timing and cellular heterogeneity of such changes will provide deeper insights into glaucoma pathophysiology.

Metabolic regulation is an important aspect of myeloid-derived cell polarity and function. Resident microglia can exhibit similar M1/M2 phenotypes to peripheral macrophages, the former representing an activated, pro-inflammatory phenotype and the latter a resting, anti-inflammatory phenotype [75]. An M1 state is consistent with aerobic glycolysis, where metabolic resources can be directed towards cell proliferation and activation, and where the ROS generated for phagocytic clearance will not interfere with and uncouple electron transport [76]. An M2 polarisation state is consistent with increased glucose metabolism and mitochondrial biogenesis [77]. Microglia in our dataset did not conform to either phenotype; we observed increased expression in glycolysis genes (including *Hif1a*), but also an upregulation of OXPHOS genes. In fact, the microglia exhibited increased metabolic upregulation from multiple energy sources. RNA sequencing of microglia in neurodegenerative disease has shown that microglial exhibit more nuanced states than these simple M1 and M2 polar opposites [14, 72, 78–80]. There is also evidence to suggest that dysregulation of metabolism and accumulation of mt-DNA mutations within microglia may itself be a trigger for microglial activation in neurodegenerative disease [81]. Dysregulated metabolism in microglia may also impair their ability to mitigate neuroinflammation. *Trem2*^*−/−*^ 5XFAD mice (an Alzheimer’s disease model) show impaired glycolysis, reduced ATP production, and increased autophagy in microglia. This disruption to metabolism may contribute to the reduced microglial phagocytosis observed in *Trem2*^*−/−*^ 5XFAD mice. Microglia in aged mice also display upregulated oxidative phosphorylation and utilisation of ketone energy sources, which may represent a stress response or loss of transcriptional regulation [82]. The dysregulation of metabolism seen in D2 microglia may be indicative of metabolic stress which may perturb microglial responses to neuroinflammation. Therapeutic strategies that target microglia metabolism may be valid targets for glaucoma treatment.

## Conclusions

This study identifies alterations in mitochondrial gene expression as well as changes to phagocytosis, inflammatory, and sensome pathways in microglia prior to optic nerve degeneration in DBA/2 J glaucoma. Out of this complex set of changes, the TREM/TYROBP signalling network emerged as a potential master regulator of microglial responses to elevated IOP. This type of response is observed in other neurodegenerative diseases where homeostatic functions of microglia are suppressed by inhibition of TYROBP signalling. This occurred in the absence of a clear pro-inflammatory response by microglia, suggesting that early pro-inflammatory signals may largely derive from infiltrating monocyte-like cells in this model of glaucoma. These data shed new light on the early myeloid changes in the ONH following periods of ocular hypertension and offer novel targets for treatment and further mechanistic exploration.

## Supplementary information

**Additional file 1.**

**Additional file 2.**

**Additional file 3.**

**Additional file 4.**

## Data Availability

All data generated or analysed during this study are included in this published article [and its supplementary information files].

## Abbreviations

ACUC: Animal Care and Use Committee
D2: DBA/2 J mouse
DE: Differentially expressed
FACS: Fluorescent activated cell sorting
FDR: False discovery rate
HC: Hierarchical clustering
IBC: Institutional Biosafety Committee
IOP: Intraocular pressure
ONH: Optic nerve head
PPD: Paraphenylenediamine
RAD: Radiation treated / irradiation therapy
